# Translation and Psychometric Validation of the Amharic eHealth Literacy Questionnaire: Cross-Sectional Study

**DOI:** 10.2196/87814

**Published:** 2026-07-16

**Authors:** Gesine Meng, Nuhamin Tekle Gebre, Abel Shita, Eyerusalem Getachew, Alemnew Destaw, Nicola Cera Schroeder, Marcus Heise, Kay Brauer, Patrick Jahn, Eva Johanna Kantelhardt, Adamu Addissie, Muluken Gizaw, Sefonias Getachew, Eric Sven Kroeber

**Affiliations:** 1 Global and Planetary Health Working Group, Institute of Medical Epidemiology, Biometrics, and Informatics, Center of Health Sciences, Medical Faculty, Martin Luther University Halle-Wittenberg, Magdeburger Str. 8, Halle, Saxony-Anhalt, 06112, Germany, +49 3455573586; 2 Cicely Saunders Institute, Florence Nightingale Faculty of Nursing, Midwifery and Palliative Care, King's College London, London, England, United Kingdom; 3 Department of Epidemiology and Biostatistics, School of Public Health, Addis Ababa University, Addis Ababa, Ethiopia; 4 Department of Gynecology, University Hospital Halle, Martin Luther University Halle-Wittenberg, Halle, Saxony-Anhalt, Germany; 5 Institute of General Practice and Family Medicine, Medical Faculty, Martin Luther University Halle-Wittenberg, Halle, Saxony-Anhalt, Germany; 6 Health Service Research Group, Center of Health Sciences, Department of Internal Medicine, Medical Faculty, Martin Luther University Halle-Wittenberg, Halle, Saxony-Anhalt, Germany; 7 Department of Internal Medicine, Center of Health Sciences, Health Service Research Group, Medical Faculty, Martin Luther University Halle-Wittenberg, Halle, Saxony-Anhalt, Germany

**Keywords:** eHealth Literacy Questionnaire, eHLQ, validation, digital health, eHealth, eHealth literacy, computer literacy, confirmatory factor analysis, Ethiopia

## Abstract

**Background:**

eHealth interventions have demonstrated potential to address challenges related to health and the health care system in low- and middle-income countries. To effectively leverage eHealth in supporting health care in Ethiopia, the assessment and development of the eHealth literacy of patients are essential.

**Objective:**

This study aimed to translate the eHealth Literacy Questionnaire (eHLQ) to Amharic and assess its psychometric properties.

**Methods:**

A systematic process of translation, including forward and backward translation, expert review, and cognitive interviews, was applied. Then, a cross-sectional questionnaire-based study using a convenience sample (N=300) of patients with internet access in the primary health care level between January and March 2025 in the capital and a larger city of Ethiopia was conducted. Internal consistency was assessed using Cronbach α and McDonald ω. The factor structure was assessed using confirmatory factor analysis. Convergent and discriminant validity were examined by calculating Spearman correlations between each eHLQ scale and the total score of the eHealth Literacy Scale (eHEALS).

**Results:**

A total of 300 participants were included in the analysis. The mean age was 30.4 (SD 6.8, range 18‐55) years, and 69.7% (209/300) were women. Internal consistency was acceptable for all scales (Cronbach α=0.72‐0.91; McDonald ω=0.79‐0.96), except for Scale 4 (α=0.62; ω=0.70). The 7-factor model showed a satisfactory fit, with a comparative fit index of 0.97, Tucker-Lewis index of 0.97, and standardized root mean square residual of 0.07. Factor loadings exceeded 0.40 for all items except one. Strong correlations between Scales 1 to 3 and eHEALS (range *r*=0.69‐0.74) supported convergent validity, whereas moderate correlations between Scales 5 to 7 and eHEALS (range *r*=0.66‐0.67) indicated limited discriminant validity.

**Conclusions:**

The Amharic eHLQ demonstrated generally satisfying psychometric properties and can be considered a valid tool for assessing eHealth literacy among patients with internet access in Ethiopia, marking the first validation of the eHLQ in sub-Saharan Africa. Future studies could provide additional evidence to substantiate the psychometric robustness of Scale 4 (“feeling safe and in control”). Overall, the Amharic eHLQ can support the development of tailored eHealth interventions in Ethiopia.

## Introduction

The World Health Organization’s (WHO) Global Strategy on Digital Health 2020 to 2025 promotes the use of sustainable, people-centered digital technologies to strengthen health systems [1]. eHealth refers to “cost-effective and secure use of information and communications technologies in support of health and health-related fields, including health care services, health surveillance, health literature, and health education, knowledge and research” [2]. In recent years, the uptake of electronic medical records, electronic health records, telemedicine, eLearning, and especially mobile health has increased globally [3]. Ethiopia’s Ministry of Health demonstrates commitment to digitalizing health care to improve equity, quality, and timeliness of care, as reflected in policy documents such as the Digital Health Blueprint [4-6]. Patients are envisioned as active users of digital health services, for example, by accessing personal health records or using health apps [6].

A systematic review highlights the potential of eHealth to address health system challenges in Ethiopia, for example, through mobile health such as SMS text messaging that supports treatment adherence and patient follow-up [7]. Moreover, studies report willingness among patients to use mobile apps and telemedicine [8-10]. However, actual usage remains low [8,9] due to multiple barriers, including organizational, technological, individual, economic, and policy dimensions [7,11-14]. Addressing these barriers is essential to ensure sustainable and equitable access to eHealth services across the country.

Despite economic growth [15], Ethiopia’s digitalization remains low compared with other African countries [16]. Mobile phone ownership was 59% in 2019 [16] and internet penetration, while steadily increasing, was estimated at 21.3% in 2025 [17,18]. Access to digital technologies is unevenly distributed, especially along urban-rural and gender lines [19]. For example, men living in rural areas were about 5 times more likely to own a mobile phone compared to their wives [20]. Beyond connectivity and device access, limited digital and eHealth literacy remains a barrier to the effective use of eHealth services in Ethiopia [12,14,21-24].

Multiple theoretical frameworks have been proposed to conceptualize eHealth literacy. Norman and Skinner [25] introduced the Lily model in 2006 as a combination of 6 literacies: traditional literacy and numeracy, information literacy, media literacy, health literacy, computer literacy, and science literacy. Based on this model, the 8-item eHealth Literacy Scale (eHEALS) was developed, validated, and used across numerous languages and cultural backgrounds [26], including Japanese [27], Persian [28], and Amharic, an official working language in Ethiopia [29,30]. However, given the fundamental evolution of the internet and the rapid advancement of eHealth technologies since 2006, the eHEALS has been criticized for not comprehensively capturing the concept of contemporary eHealth literacy [26,31,32].

To account for evolving digital health environments, the Lily model has been extended, alongside the development of additional frameworks [31,33-35]. One such framework is the eHealth Literacy Framework (eHLF), developed by Norgaard et al [35] in 2015 through concept mapping with patients, health professionals, and digital health experts in Denmark and the United Kingdom. The eHLF comprises 3 areas: “individual,” “interaction,” and “system.” The area “individual” includes two domains: (1) ability to process information and (2) engagement in own health; “interaction” comprises three domains: (3) ability to engage actively with digital services, (4) feeling safe and in control, and (5) motivation to engage with digital services; “system” includes two domains: (6) having access to systems that work and (7) digital services that suit individual needs [35]. Building on the eHLF, Kayser et al [36] developed the eHealth Literacy Questionnaire (eHLQ) in 2018 [26,36,37]. The eHLQ consists of 35 items distributed across 7 scales, each corresponding to one eHLF domain: (1) using technology to process health information (5 items), (2) understanding of health concepts and language (5 items), (3) ability to actively engage with digital services (5 items), (4) feeling safe and in control (5 items), (5) motivated to engage with digital services (5 items), (6) access to digital services that work (6 items), and (7) digital services that suit individual needs (4 items) [36]. The eHLQ was initially validated in Danish [36] and English [38] and has been translated and validated in multiple languages, including Mandarin [39], Norwegian [40], Spanish and Catalan [41], Dutch [42], Swedish [43], Serbian [44], and Arabic [45]. Although the eHLQ can be applied within the Ophelia (Optimizing Health Literacy and Access) process [46], it was developed as an independent measurement instrument and is not inherently part of, nor restricted to, this process or its WHO-related applications [36,46,47].

eHealth literacy is associated with greater health-promoting behavior, better health attitudes and knowledge, and improved medication adherence [48-50]. Low levels are linked to poorer health outcomes [51]. Understanding eHealth literacy of patients is essential to gain insight into their experiences with digital health services, to provide targeted support to improve access and use, and to inform the development of digital health technologies [36,52].

In Ethiopia, a study that applied the eHEALS among patients who were chronically ill in 2020 found a mean eHEALS score of 24.6 [22,53]. Additionally, studies among undergraduate nursing students in 2020 and medical and health science students in 2021 reported mean eHEALS scores of 25.2 and 28.7, respectively [54,55]. Determinants of eHealth literacy include knowledge and use of eHealth information sources, perceived usefulness, internet access, and gender [56]. To the best of our knowledge, no eHealth literacy instrument other than eHEALS has been translated into and validated in Amharic.

The aim of this study is to translate the eHLQ into Amharic and evaluate its psychometric properties by providing evidence for structural, convergent, and discriminant validity as well as its reliability by means of internal consistency.

## Methods

### Study Design and Study Settings

We conducted a facility-based cross-sectional study and collected data from January 21 to March 10, 2025, at Churchill Health Center in Addis Ababa and Cheleleka Health Center in Bishoftu. Addis Ababa, Ethiopia’s capital, is the urban center of the country with about 6 million people living in the metropolitan area [57]. Bishoftu is a town of about 200,000 inhabitants located 50 km southeast of Addis Ababa [58]. The Ethiopian public health system follows a 3-tiered structure, with primary hospitals, health centers, and health posts at the primary level; general hospitals at the secondary level; and specialized hospitals at the tertiary level [59]. Both study sites are primary-level health care facilities. This cross-sectional study was reported in accordance with the STROBE (Strengthening the Reporting of Observational Studies in Epidemiology) guidelines.

### Questionnaire

For data collection, we used a questionnaire comprising the Amharic eHLQ, the Amharic eHEALS [29], items on internet and technology use, and sociodemographic information (age, gender, education, occupation, and income). Participants completed the eHLQ using a 4-point Likert scale (1=strongly agree to 4=strongly disagree) and the eHEALS using a 5-point Likert scale (1=strongly agree to 5=strongly disagree). While the eHEALS provides a global score, the eHLQ does not, and its scales are interpreted independently.

### Translation of the eHLQ

We obtained a license from Swinburne University, Australia, to translate the English eHLQ into Amharic. We applied the Swinburne Translation Integrity Procedure to ensure measurement equivalence [60]. Two native Amharic speakers fluent in English conducted the forward translation: one prepared the initial draft, and the other reviewed it. After discussing discrepancies, the version was jointly reviewed with a member of the eHLQ development team. A native Amharic speaker with high English proficiency, who had not seen the original questions, conducted the back translation. The final consensus meeting, chaired by a member of the eHLQ development team, involved all translators, bilingual team members from Ethiopia, and team leaders from Ethiopia and Germany. The group reviewed all items until agreement was reached. Four items (6, 9, 23, and 26) required detailed discussion because of translation challenges. For item 25, which is about using technology to organize health information, the local team raised concerns about contextual appropriateness, as organizing health information is typically conducted by health professionals, not patients. No item was removed or significantly modified to maintain the scale’s psychometric integrity.

### Pretests and Tool Quality Maintenance

One trained data collector conducted cognitive interviews with 9 Amharic speakers in Addis Ababa to identify potential challenges in item interpretation. The sample aimed to represent demographic variety and included patients, office workers, and a nurse from Churchill Health Center in Addis Ababa, as well as older adults personally known to the data collector. Ages ranged from 29 to 62 years, and educational levels ranged from finishing 10th grade to master’s degree. We did not specify internet access as an inclusion criterion for the cognitive interviews, as we initially intended to validate the questionnaire in the general population. All participants provided written informed consent, and the interviews were audio-recorded with permission. Participant feedback was collected using the think-aloud method [61] and analyzed using inductive coding.

Challenges emerged across items of all scales, mainly related to translation clarity, technical terminology, contextual relevance, and conceptual interpretation. *Translation issues:* Item 23 was unclear because the translation of “work together” implied active collaboration. *Technical terminology:* Participants, particularly individuals with limited digital exposure, found terms such as “digital health technologies” difficult to interpret consistently (eg, items 7, 8, and 10). *Contextual relevance and applicability:* Limited familiarity with technology and digital health technologies in particular caused challenges (eg, items 8, 9, 18, and 23). *Conceptual interpretation:* Some participants struggled with vague references (eg, “people who are supposed to”) in items 1 and 3. Item 21 was often interpreted as referring to external body functions (eg, movement of the hand) rather than internal functions. Item 26 raised uncertainty regarding whether “measurements” referred only to digital tools or included manual methods. Furthermore, a few items were perceived as repetitive, and older participants reported greater difficulty completing the questionnaire.

Based on the pretest findings, adjustments were implemented. We revised the translation of “work together” in item 23 to better reflect the idea of system coordination in the context of different technologies. Furthermore, we trained data collectors to use the eHLQ introductory information, thereby minimizing interpretation challenges and clarifying item intent when needed. Finally, we refined the inclusion criteria, adding smartphone ownership and access to the internet, to ensure that participants had access to technology and the internet to enable meaningful engagement with the questionnaire.

### Data Collection

We recruited 2 experienced Ethiopian data collectors with backgrounds in public health and medical care and provided a 1-day training, which included a comprehensive review of the questionnaire and a practical session to pilot data collection procedures. Data were collected in a REDCap database [62].

### Study Participants and Sample Size

We recruited participants using convenience sampling at Churchill Health Center in Addis Ababa and Cheleleka Health Center in Bishoftu. Inclusion criteria included having experience as a patient in the Ethiopian health care system, being 18 years of age or older, the ability to read a text in the Amharic language, ownership of a smartphone, and access to the internet. Smartphone ownership and internet access were chosen as the eHLQ measures users’ competencies and perceptions related to digital health services. Informed by the cognitive interviews, these criteria were intended to support meaningful item interpretation and reduce construct-irrelevant variance related to lack of digital access [63]. Churchill Health Center in Addis Ababa and Cheleleka Health Center in Bishoftu were selected as recruitment sites because both are primary health care facilities in urban settings where Amharic is widely spoken and access to the internet and smartphones is comparatively high [64].

Based on the recommendation by Comrey and Lee [65], we aimed to recruit at least 300 participants.

During data collection, 356 individuals were approached with support from health care staff. Of these, 54 were not eligible: 31 did not own a smartphone, 5 did not have internet access, and 18 had neither. This left 302 participants. In addition, participants with more than 50% missing data were excluded (n=2). The final analytic sample comprised 300 participants. Missing data were handled using listwise deletion for the confirmatory factor analysis (CFA) with the weighted least squares mean and variance–adjusted (WLSMV) estimator and pairwise deletion for Spearman correlations.

### Data Analysis

We conducted data analysis using R (version 4.5.0; R Foundation for Statistical Computing) [66] and Mplus (version 8.11; Muthén and Muthén) [67]. Structural equation modeling was performed with the *lavaan* package [68]. Descriptive statistics were used to summarize participants’ demographics. For continuous variables, we reported means (SD) or medians (IQR). For categorical variables, we presented absolute and relative frequencies. Mean scores were calculated for each eHLQ scale, with lower scores indicating stronger agreement. Floor and ceiling effects in items were defined as ≥15% of responses in the lowest or highest category [69].

Psychometric evaluation followed the Standards for Educational and Psychological Testing [63], gathering evidence from multiple sources. We examined structural, convergent, and discriminant validity as well as internal consistency. As no causal effect estimates were planned, no adjustment for potential confounders was performed. Because the Amharic eHLQ is a translation of a prestructured questionnaire, we computed a CFA using the WLSMV estimator, which is suitable for ordinal data [70,71]. Missing data were handled through listwise deletion, resulting in 251 observations being included in the CFA.

Standardized factor loadings above 0.4 were considered acceptable, following the cutoff applied in the Swedish validation study [43]. *R*² values were calculated to estimate the proportion of variance explained by each factor.

We assessed model fit using the following Goodness-of-Fit Indices: chi-square (*χ*^2^) [72], comparative fit index (CFI) [73], Tucker-Lewis index (TLI) [74], root mean square error of approximation (RMSEA) [75], and standardized root mean square residual (SRMR) [72]. Established cutoff values are CFI and TLI >0.95, RMSEA <0.06, and SRMR <0.08 [72,75]. Compared to other fit indices such as RMSEA and CFI, the SRMR is considered more stable across different estimation methods [76,77]. However, robust cutoffs for fit indices using the WLSMV estimator are not yet available [78,79]. Therefore, model fit was evaluated in comparison with prior CFA findings from translations of the eHLQ (Multimedia Appendix 1) and considered acceptable if within the reference ranges CFI ≥0.93, TLI ≥0.92, SRMR ≤0.09, and RMSEA ≤0.09.

Scale intercorrelations were assessed using Spearman correlations between the mean scores of the eHLQ scales, with 95% CIs obtained via bootstrapping.

Given the high interfactor correlations reported in previous studies, we explored 3 alternative model structures: a single-factor model to assess the distinctiveness of the factors and a second-order and a bifactor model to evaluate a potential higher order or general factor. Model comparisons were performed using chi-square difference tests.

Internal consistency was assessed using Cronbach α and McDonald ω values, with 95% CIs obtained via bootstrapping. Values ≥0.70 for both tests were considered acceptable [80,81].

To assess convergent and discriminant validity, we administered the Amharic eHEALS [29] as a reference instrument. The expected pattern of correlations derives from the conceptual mapping of the eHLF, which underpins the eHLQ, to the Norman and Skinner Lily model, which underpins the eHEALS [25,35,36,53]. As shown by Norgaard et al [35], the 6 literacies of the Lily model are distributed across the first 3 domains of the eHLF, which are operationalized in eHLQ Scales 1 to 3. The remaining 4 eHLF domains have no equivalent in the Norman and Skinner model and are operationalized in eHLQ Scales 4 to 7 [35]. Hence, we expected convergent validity with the eHEALS for eHLQ scales 1 to 3 and discriminant validity for eHLQ Scales 4 to 7. Total scores of eHEALS were correlated with scores from each eHLQ scale using Spearman correlation with 95% CIs. We interpreted large correlations (ρ≥0.7) for Scales 1 to 3 as evidence of convergent validity, whereas weak correlations (ρ<0.4) for Scales 4 to 7 as evidence of discriminant validity [82].

### Ethical Considerations

This study received ethical approval from the School of Public Health, Addis Ababa University (ref number EP&BI/274/2025) and the Medical Faculty of Martin Luther University Halle-Wittenberg, Germany (processing number 2024‐223). All participants provided written informed consent. Data were collected using REDCap [62]. Participants’ privacy and confidentiality were maintained throughout the study. All data were recorded anonymously, and no identifying information was stored. No compensation was provided for participation.

## Results

### Description of the Sample

The mean age of participants was 30.4 (SD 6.8, range 18‐55) years (Table 1). Of the 300 participants, 209 (69.6%) were women, and 271 (90.3%) lived in an urban area. Regarding education, 59 (19.7%) had completed grade 12, whereas 121 (40.3%) had attained higher education. More than 9 out of 10 participants (276/300, 92%) used technology (eg, smartphones) at least once a day, and 230 (76.7%) used the internet at least once a day.

**Table 1.** Participant’s characteristics: demographics and technology usage (N=300).

Sociodemographic characteristics	Participants
Age (y), mean (SD)	30.4 (6.8)
Range: min-max	18‐55
Gender, n (%)	
Men	91 (30.3)
Women	209 (69.6)
Residential area, n (%)	
Urban	271 (90.3)
Semiurban	25 (8.3)
Rural	2 (0.7)
Missing	2 (0.7)
Education, n (%)	
Below grade 8	44 (14.7)
Grades 8 to 11	76 (25.3)
Finished grade 12	59 (19.7)
Higher education	121 (40.3)
Occupation, n (%)	
Homemaker	76 (25.3)
Government employee	68 (22.7)
Nongovernment employee	100 (33.3)
Working but not employee	31 (10.3)
Not working	25 (8.3)
Monthly household income (Ethiopian birr), median (IQR)	10,000 (6000‐15,000)
Range: min-max	0‐90,000
Technology and internet use, n (%)	
Regular technology use (smartphone, laptop, computer, or tablet)	
At least once a day	276 (92)
Once every few days	5 (1.7)
Once a week	14 (4.7)
Once in the last 3 months	4 (1.3)
Less	1 (0.3)
Regular internet use, n (%)	
At least once a day	230 (76.7)
Once every few days	28 (9.3)
Once a week	30 (10)
Once in the last 3 months	11 (3.7)
Less	1 (0.3)

### Descriptive Statistics eHLQ

Mean scores on the eHLQ scales ranged from 2.0 (SD 0.4) on Scale 4 (“feeling safe and in control”) to 2.4 (SD 0.5) on Scale 2 (“understanding of health concepts and language”). Ceiling effects were observed in 6 items across different scales (items 1, 2, 3, 6, 7, and 14), and floor effects were found in 1 item (item 26; Table 2).

**Table 2.** Descriptive statistics and floor and ceiling effects of the Amharic eHLQ^a^.

Item	Mean (SD)	Median (IQR)	1–Strongly agree, n (%)	2–Agree, n (%)	3–Disagree, n (%)	4–Strongly disagree, n (%)	Missing, n (%)
Scale 1: using technology to process health information							
Overall	2.3 (0.5)						
eHLQ7		2 (2-3)	48 (16)^b^	171 (57)	77 (25.7)	2 (0.7)	2 (0.7)
eHLQ11		2 (2-3)	33 (11)	157 (52.3)	103 (34.3)	6 (2)	1 (0.3)
eHLQ13		2 (2-3)	39 (13)	178 (59.3)	79 (26.3)	2 (0.7)	2 (0.7)
eHLQ20		2 (2-3)	27 (9)	168 (56)	89 (29.7)	14 (4.7)	2 (0.7)
eHLQ25		2 (2-3)	19 (6.3)	136 (45.3)	135 (45)	8 (2.7)	2 (0.7)
Scale 2: understanding of health concepts and language							
Overall	2.4 (0.5)						
eHLQ5		2 (2-3)	43 (14.3)	171 (57)	84 (28)	1 (0.3)	1 (0.3)
eHLQ12		2 (2-3)	26 (8.7)	164 (54.7)	107 (35.7)	2 (0.7)	1 (0.3)
eHLQ15		2 (2-3)	25 (8.3)	143 (47.7)	125 (41.7)	5 (1.7)	2 (0.7)
eHLQ21		2 (2-3)	23 (7.7)	161 (53.7)	107 (35.7)	8 (2.7)	1 (0.3)
eHLQ26		3 (3-4)	14 (4.7)	45 (15)	147 (49)	94 (31.3)^b^	0 (0)
Scale 3: ability to actively engage with digital services							
Overall	2.2 (0.6)						
eHLQ4		2 (2-3)	44 (14.7)	165 (55)	88 (29.3)	2 (0.7)	1 (0.3)
eHLQ6		2 (2-2)	48 (16)^b^	181 (60.3)	69 (23)	1 (0.3)	1 (0.3)
eHLQ8		2 (2-3)	39 (13)	150 (50)	107 (35.7)	3 (1)	1 (0.3)
eHLQ17		2 (2-3)	22 (7.3)	154 (51.3)	108 (36)	14 (4.7)	2 (0.7)
eHLQ32		2 (2-3)	17 (5.7)	173 (57.7)	96 (32)	10 (3.3)	4 (1.3)
Scale 4: feeling safe and in control							
Overall	2.0 (0.4)						
eHLQ1		2 (1-2)	108 (36)^b^	169 (56.3)	22 (7.3)	1 (0.3)	0 (0)
eHLQ10		2 (2-3)	32 (10.7)	184 (61.3)	78 (26)	5 (1.7)	1 (0.3)
eHLQ14		2 (2-2)	46 (15.3)^b^	181 (60.3)	71 (23.7)	2 (0.7)	0 (0)
eHLQ22		2 (2-2)	44 (14.7)	193 (64.3)	58 (19.3)	2 (0.7)	3 (1.0)
eHLQ30		2 (2-2)	36 (12)	212 (70.7)	48 (16)	4 (1.3)	0 (0)
Scale 5: motivated to engage with digital services							
Overall	2.2 (0.5)						
eHLQ2		2 (2-2)	60 (20)^b^	173 (57.7)	67 (22.3)	0 (0)	0 (0)
eHLQ19		2 (2-3)	34 (11.3)	187 (62.3)	63 (21)	13 (4.3)	3 (1)
eHLQ24		2 (2-3)	21 (7)	191 (63.7)	81 (27)	6 (2)	1 (0.3)
eHLQ27		2 (2-2)	30 (10)	202 (67.3)	63 (21)	3 (1)	2 (0.7)
eHLQ35		2 (2-2)	29 (9.7)	196 (65.3)	69 (23)	5 (1.7)	1 (0.3)
Scale 6: access to digital services that work							
Overall	2.4 (0.4)						
eHLQ3		2 (2-3)	45 (15)^b^	158 (52.7)	80 (26.7)	16 (5.3)	1 (0.3)
eHLQ9		2 (2-3)	31 (10.3)	149 (49.7)	112 (37.3)	5 (1.7)	3 (1)
eHLQ16		3 (2-3)	15 (5)	107 (35.7)	159 (53)	18 (6)	1 (0.3)
eHLQ23		2 (2-3)	19 (6.3)	143 (47.7)	132 (44)	6 (2)	0 (0)
eHLQ29		3 (2-3)	14 (4.7)	127 (42.3)	130 (43.3)	27 (9)	2 (0.7)
eHLQ34		2 (2-3)	25 (8.3)	180 (60)	91 (30.3)	3 (1)	1 (0.3)
Scale 7: digital services that suit individual needs							
Overall	2.3 (0.6)						
eHLQ18		2 (2-3)	27 (9)	169 (56.3)	83 (27.7)	19 (6.3)	2 (0.7)
eHLQ28		2 (2-3)	32 (10.7)	181 (60.3)	75 (25)	9 (3)	3 (1)
eHLQ31		2 (2-3)	17 (5.7)	185 (61.7)	85 (28.3)	11 (3.7)	2 (0.7)
eHLQ33		2 (2-3)	26 (8.7)	186 (62)	71 (23.7)	11 (3.7)	6 (2)

a eHLQ: eHealth Literacy Questionnaire.

b ≥15% (n=45) selected the lowest or highest response (floor/ceiling effect).

### Psychometric Properties

We fitted a 7-factor model to the present data (Table 3). Values of SRMR, CFI, and TLI (0.07, 0.97, and 0.97, respectively) aligned with values of prior validation studies (Multimedia Appendix 1), indicating a satisfactory model fit. The RMSEA (0.10, 90% CI 0.09‐0.10) was slightly higher than values reported in prior validation studies (Multimedia Appendix 1). In addition, 34 out of 35 items showed loadings above 0.4 (Multimedia Appendix 2). One exception was item 1 of Scale 4 (“feeling safe and in control”) (λ=.18; *P*=0.01; *R*^2^=0.03). Thirty out of 35 items showed loadings above 0.5. All factor loadings had *P*=.01 (Multimedia Appendix 2).

Scale intercorrelations ranged from 0.36 (95% CI 0.23‐0.47) between Scale 4 and Scale 7 to 0.84 (95% CI 0.79‐0.88) between Scale 3 and Scale 7 and 0.84 (95% CI 0.80‐0.88) between Scale 5 and Scale 7. Most correlations were high, with the majority being above 0.7 (Table 4).

**Table 3.** Goodness-of-fit indices for the 7-factor, single-factor, and second-order models of the Amharic eHealth Literacy Questionnaire.

Model	SRMR^a^	CFI^b^	TLI^c^	RMSEA^d^ (90% CI)	Chi-square (*df*)	*P* value
7-factor model	0.07	0.97	0.97	0.10 (0.09‐0.10)	1790.5 (539)	<.001
Single-factor model	0.08	0.96	0.96	0.10 (0.10‐0.11)	2275.5 (560)	<.001
Second-order factor model	0.07	0.96	0.96	0.10 (0.10‐0.10)	2202.2 (553)	<.001

a SRMR: standardized root mean residual.

b CFI: comparative fit index.

c TLI: Tucker-Lewis index.

d RMSEA: root mean square error of approximation.

**Table 4.** Spearman correlations (95% CIs) between the Amharic eHEALS^a^ total score and Amharic eHLQ^b^ scale scores and scale intercorrelations between eHLQ scale means.

eHLQ scale	eHEALS	Scale 1	Scale 2	Scale 3	Scale 4	Scale 5	Scale 6
Scale 1: using technology to process health information	0.70 (0.64‐0.75)						
Scale 2: understanding of health concepts and language	0.69 (0.62‐0.74)	0.77 (0.71‐0.82)					
Scale 3: ability to actively engage with digital services	0.74 (0.69‐0.79)	0.82 (0.77‐0.87)	0.77 (0.71‐0.82)				
Scale 4: feeling safe and in control	0.41 (0.31‐0.50)	0.45 (0.34‐0.56)	0.34 (0.22‐0.45)	0.41 (0.30‐0.52)			
Scale 5: motivated to engage with digital services	0.67 (0.60‐0.67)	0.81 (0.76‐0.86)	0.71 (0.63‐0.77)	0.81 (0.76‐0.86)	0.43 (0.32‐0.54)		
Scale 6: access to digital services that work	0.66 (0.60‐0.66)	0.79 (0.73‐0.84)	0.67 (0.59‐0.74)	0.78 (0.72‐0.83)	0.50 (0.41‐0.60)	0.72 (0.65‐0.78)	
Scale 7: digital services that suit individual needs	0.67 (0.61‐0.73)	0.78 (0.71‐0.83)	0.73 (0.66‐0.78)	0.84 (0.79‐0.88)	0.36 (0.23‐0.47)	0.84 (0.80‐0.88)	0.71 (0.64‐0.77)

a eHEALS: eHealth Literacy Scale.

b eHLQ: eHealth Literacy Questionnaire.

The estimation of the bifactor model was unsuccessful due to nonconvergence and was therefore excluded from further analysis. The results of the single-factor and second-order factor models are presented in Table 3. The chi-square difference test to evaluate differences of the second-order factor model and the 7-factor model rejected the corresponding null hypothesis (χ^2^_14_=411.78, *P*<.001), indicating that the more complex model, including 7 first-order factors and a second-order factor, explains the data best.

Internal consistency was acceptable to excellent across most scales, with Cronbach α ranging from 0.72 (95% CI 0.63‐0.77) to 0.91 (95% CI 0.89‐0.93) and McDonald ω from 0.79 (95% CI 0.72‐0.83) to 0.96 (95% CI 0.94‐0.97). Only Scale 4 (“feeling safe and in control”) showed a lower Cronbach α of 0.62 (95% CI 0.51‐0.70) but an acceptable McDonald ω of 0.70 (95% CI 0.61‐0.77). For the total score, representing the second-order factor of eHealth literacy, Cronbach α was 0.96 (95% CI 0.96‐0.97) and McDonald ω was 0.97 (95% CI 0.96‐0.98) (Table 5).

**Table 5.** Internal consistency of the Amharic eHLQ^a^.

Model	Cronbach α (95% CI)	McDonald ω (95% CI)
Scale 1: using technology to process health information	0.87 (0.84‐0.90)	0.91 (0.88‐0.94)
Scale 2: understanding of health concepts and language	0.81 (0.74‐0.83)	0.86 (0.80‐0.89)
Scale 3: ability to actively engage with digital services	0.90 (0.88‐0.92)	0.94 (0.90‐0.96)
Scale 4: feeling safe and in control	0.62 (0.51‐0.70)	0.70 (0.61‐0.77)
Scale 5: motivated to engage with digital services	0.89 (0.86‐0.91)	0.92 (0.89‐0.93)
Scale 6: access to digital services that work	0.72 (0.63‐0.77)	0.79 (0.72‐0.83)
Scale 7: digital services that suit individual needs	0.91 (0.89‐0.93)	0.96 (0.94‐0.97)
eHLQ (total)	0.96 (0.96‐0.97)	0.97 (0.96‐0.98)

a eHLQ: eHealth Literacy Questionnaire.

The mean of the Amharic eHEALS total scores was 20.1 (SD 6.9), with observed total scores ranging from 8 to 40. We found strong positive correlations between the Amharic eHEALS total scores and Scales 1, 2, and 3 of the eHLQ (ρ=0.70, ρ=0.69, and ρ=0.74, respectively), supporting convergent validity (Table 4). Although the correlation with Scale 2 was slightly (ρ=0.01) below the predefined cutoff of ρ=0.70, we cautiously interpreted it as evidence of convergent validity. Correlations between the Amharic eHEALS total score and Scales 4 to 7 of the Amharic eHLQ ranged from ρ=0.41 (95% CI 0.31‐0.50) for Scale 4 (“feeling safe and in control”) to ρ=0.67 (95% CI 0.60‐0.67) for Scale 5 (“ability to actively engage with digital services”) and ρ=0.67 (95% CI 0.61‐0.73) for Scale 7 (“digital services that suit individual needs”). With the correlations of Scales 5, 6, and 7 exceeding 0.6, reflecting moderate associations, our findings provide only limited evidence for discriminant validity between the Amharic eHLQ and the Amharic eHEALS (Table 4).

## Discussion

### Principal Findings

This study aimed to translate the eHLQ into Amharic and evaluate its psychometric properties. We assessed structural, convergent, and discriminant validity, as well as internal consistency. Our results showed satisfactory fit for a 7-factor model, robust internal consistency in line with expectations for comparatively short scales [83], acceptable evidence for convergent validity, and mixed evidence for discriminant validity.

### Descriptive Statistics

Six items showed ceiling effects, and 1 item showed a floor effect, indicating limited differentiation at the extremes for only a few items, while the overall response distribution was adequate. In comparison, the Swedish eHLQ found ceiling effects in most items, possibly reflecting greater confidence in digital health skills and systems among the Swedish sample, which may be partly due to Sweden’s highly digitalized health care [43,49,84].

With a scale ranging from 1 (strongly agree) to 4 (strongly disagree), mean scores between 2.0 and 2.4 indicate an average to a slightly below-average level of eHealth literacy across the eHLQ scales. The highest mean score was observed for Scale 2 (“understanding of health concepts and language”) and Scale 6 (“access to digital services that work”), suggesting greater disagreement on these scales. Since Scale 2 assesses aspects of health literacy [35], this may reflect low health literacy levels, as previously reported in Ethiopia [85]. Disagreement on Scale 6 may reflect barriers to accessing technology and digital health services. For example, as described by the local research and clinical teams, patients have only limited access to their personal health data [6].

We observed the lowest mean score for Scale 4 (“feeling safe and in control”), suggesting that most participants felt safe and in control regarding data storage and authorized access. However, cognitive interview results and follow-up research team discussions suggested difficulty in the comprehensibility of Scale 4 items in the local context, which might be reflected in the comparably less favorable psychometric properties, as discussed below.

### Psychometric Properties

The CFA supported the 7-factor structure of the Amharic eHLQ. The SRMR, CFI, and TLI indicated an overall satisfactory model fit, as they fell within the range of values from previous validation studies (Multimedia Appendix 1). The slightly elevated RMSEA was not considered critical, particularly because the SRMR is regarded as more robust with the WLSMV estimator [76].

All factor loadings showed exploratory *P*=.01 (Multimedia Appendix 2). Item 1 of Scale 4 (“feeling safe and in control”) showed the only loading below 0.4 (*R*^2^=0.03), suggesting the limited representation of the underlying factor. The item addresses the certainty that only authorized individuals use one’s health care data. According to the local team, health data are typically managed by health care professionals rather than patients, which may have made the item seem irrelevant to participants. Difficulties in interpreting the phrase “who is supposed to use it,” noted during pretests, may also have contributed to the low loading. Overall, the result may reflect the influence of an external construct not captured by the fourth factor. A comparably lower loading (0.46) in the Arabic study may suggest cross-cultural measurement issues of the item [45]. As a sensitivity analysis, we repeated the CFA without item 1 (Multimedia Appendix 3). Model fit was SRMR=0.07, CFI=0.97, TLI=0.97, and RMSEA=0.09. Cronbach α of Scale 4 was 0.59 (95% CI 0.51‐0.67) and McDonald ω 0.70 (95% CI 0.42‐0.81). After extensive discussion, we decided to retain the item despite its limited performance to ensure comparability with other eHLQ versions. The findings suggest that the results from Scale 4 should be interpreted with caution. Furthermore, item 3 on Scale 6 (“access to digital services that work”) showed a comparatively low factor loading (λ=0.44, 95% CI 0.35‐0.52), consistent with findings from the Arabic and Swedish versions (0.42 and 0.35, respectively) [43,45].

In line with the Danish validation study [36], we found strong intercorrelations between eHLQ scales, indicating potential overlap of the scales. In this study, the highest correlations were observed between the means of Scales 3 and 7 as well as between Scales 5 and 7. Kayser et al [36] interpreted high interfactor correlations as reflecting domains that may lie on the same causal pathway while still being conceptually distinct [36]. In the Arabic version, high interfactor correlations led to the combination of Scales 6 and 7 [45]. We interpreted these findings as suggesting a possible general eHealth literacy factor underlying the 7 factors. The better fit of the second-order factor model supports this interpretation and may justify calculating a total eHealth literacy score.

The scales of the Amharic eHLQ demonstrated good internal consistency overall, especially given the low number of items per scale [83]. Both Cronbach α and McDonald ω exceeded 0.70 for all scales except Scale 4 (“feeling safe and in control”), indicating insufficient internal consistency for this scale. This contrasts with previous studies, where Scale 4 showed adequate values (Danish: α=0.86 [36], Swedish: *α*=0.83 [43], Arabic: ω=0.76 [45]). However, as McDonald ω for Scale 4 was 0.70 and Cronbach α exceeded 0.60, we considered the scale acceptable. Scale 6 (“access to digital services that work”) showed the second lowest values with α=0.72 (95% CI 0.63‐0.77) and ω=0.78 (95% CI 0.72‐0.83). Similarly, in the Danish and Swedish validation study, Scale 6 showed relatively low values (α=0.77 and *α*=0.82, respectively) [36,43].

Scale 4 of the Amharic eHLQ requires further attention, as it showed weaker internal consistency and included the lowest loading item (item 1). This may indicate cultural mismatches in how the construct of “feeling safe and in control” is represented or reflect limited comprehension because it is rarely addressed in the local context, as noted by the local team. Future studies should further investigate this to rule out the influence of extraneous factors or construct-irrelevant variance. Qualitative approaches may provide deeper insights into how the scale and health data security, in general, are perceived in the Ethiopian context.

The mean total score of the Amharic eHEALS in this study was 20.1 (SD 6.9), which was lower than that reported among patients with chronic conditions at the University of Gondar Comprehensive Specialized Hospital (24.6) [22]. Strong to moderate positive correlations between the eHEALS total score and most eHLQ scales indicate evidence for convergent validity but only limited evidence for discriminant validity. The limited evidence for discriminant validity is likely due to substantial scale intercorrelations. Consistent with our findings, the validation study of the Spanish translation also reported evidence of convergent validity and limited evidence of discriminant validity [41].

### Strengths, Limitations, and Future Directions

Our study has several strengths. The eHLQ is based on a robust framework [35,36] and has demonstrated stable psychometric properties across different cultural contexts [41,44,45]. We followed a rigorous translation process using the Translation Integrity Procedure, which has been successfully applied in other validation studies of the eHLQ [60]. Moreover, this study represents the first validation of the eHLQ in a low-resource country in Africa, providing valuable and novel insights into its applicability and use.

The inclusion criteria of smartphone ownership and internet access possibly limit the generalizability of our study to individuals with lower digital exposure. For this validation study, these criteria were purposefully chosen to ensure that participants had access to digital environments relevant to the construct assessed by the questionnaire. Nevertheless, our sample included a wide range of eHealth literacy levels. Beyond digital access, generalizability is further limited by convenience sampling in 2 health care facilities due to limited access to recruitment sites. Taken together, these limitations may contribute to an unbalanced sociodemographic sample, with an overrepresentation of educated women from urban areas. A context-sensitive application of the tool remains essential. Future studies should employ stratified sampling to ensure adequate representation of men, rural populations, and individuals with lower education levels.

Unlike in the original validation study [36], the Amharic eHLQ was interviewer-administered, which may have influenced participants’ responses. For instance, it could have increased the risk of socially desirable response bias or prevented participants from answering at their own pace [86,87]. To minimize this, we used experienced data collectors and provided them with 1 day of training. Data collectors were informed about the general purpose of the study but not about specific psychometric hypotheses.

Finally, to mitigate a potential unilateral perspective due to the positionality of the first author—based in Germany—and the resulting cultural differences and possible differing understandings of concepts used in the questionnaire, we ensured close collaboration with Ethiopian researchers throughout the entire study period and used a structured translation process [60,88].

Future research should focus on multiple-group CFAs and investigate metric and configural invariance to ensure that comparisons across groups are meaningful.

### Conclusion

The Amharic eHLQ can be considered a psychometrically sound instrument for assessing eHealth literacy among patients with internet access in Ethiopia, representing the first validation of the eHLQ in a sub-Saharan African context. Future studies should focus on invariance testing to ensure meaningful group comparisons and provide additional evidence to substantiate the psychometric robustness of Scale 4 (“feeling safe and in control”), as well as qualitative research on local conceptualizations and familiarity with data security. Overall, applying the Amharic eHLQ can provide valuable insights into the experiences of patients with eHealth, inform policymakers and service providers about patients’ needs, and guide the development of targeted eHealth interventions in Ethiopia.

## Supplementary material

10.2196/87814 Multimedia Appendix 1 Goodness-of-fit indices reported in previous eHealth Literacy Questionnaire validation studies.

10.2196/87814 Multimedia Appendix 2 Standardized factor loadings, SE, 95% CI, *P* value, and *R*^2^ of the 7-factor model.

10.2196/87814 Multimedia Appendix 3 Sensitivity analysis: confirmatory factor analysis without item 1 in Scale 4.

## Abbreviations

CFA: confirmatory factor analysis
CFI: comparative fit index
eHEALS: eHealth Literacy Scale
eHLF: eHealth Literacy Framework
eHLQ: eHealth Literacy Questionnaire
Ophelia: Optimizing Health Literacy and Access
RMSEA: root mean square error of approximation
SRMR: standardized root mean residual
STROBE: Strengthening the Reporting of Observational Studies in Epidemiology
TLI: Tucker-Lewis index
WHO: World Health Organization
WLSMV: weighted least squares mean and variance adjusted
