# Supplementary material for: Translation and Psychometric Validation of the Amharic eHealth Literacy Questionnaire: Cross-Sectional Study
Source: J Med Internet Res. 2026 Jul 16;28:e87814. doi: 10.2196/87814 (PMC13375083; doi:10.2196/87814)
Supplement: Multimedia Appendix 1 [file jmir-v28-e87814-s001.docx]

**Multimedia Appendix**

**Table S1.** Goodness-of-fit indices reported in previous eHLQ^a^ validation studies

| Validation Study | SRMR^b^ | CFI^c^ | TLI^d^ | RMSEA^e^ |
| --- | --- | --- | --- | --- |
| Arabic (2025) [1] | 0.06 | 0.93 | 0.92 | 0.06 |
| Catalan (2024) [2] | 0.04 | 0.98 | 0.98 | 0.07 |
| Dutch (2023) [3] | 0.09 | 0.94 | 0.93 | 0.09 |
| Serbian (2024) [4] | - | 0.95 | - | 0.05 |
| Spanish (2024) [2] | 0.04 | 0.98 | 0.98 | 0.07 |
| Swedish (2023) [5] | 0.06 | 1 | 1 | 0 |

^a^eHLQ: eHealth Literacy Questionnaire

^b^SRMR: Standardized Root Mean Residual

^c^CFI: Comparative Fit Index

^d^TLI: Tucker Lewis Index

^e^RMSEA: Root Means Square Error of Approximation

1. Al-Qerem W, Fadhil ON, Jarab A, Hammad A, Al-Asmari F, Zidan R, et al. Validation of the Arabic eHealth literacy questionnaire: A factor and Rasch analysis study. Front Public Health. 2025;13:1542477. PMID: 39991699. doi: 10.3389/fpubh.2025.1542477.

2. Hernández Encuentra E, Robles N, Angulo-Brunet A, Cullen D, del Arco I. Spanish and Catalan versions of the eHealth literacy questionnaire: Translation, cross-cultural adaptation, and validation study. J Med Internet Res. 2024;26:e49227. PMID: 38728072. doi: 10.2196/49227.

3. Poot CC, Meijer E, Fokkema M, Chavannes NH, Osborne RH, Kayser L. Translation, cultural adaptation and validity assessment of the Dutch version of the eHealth literacy questionnaire: A mixed-method approach. BMC Public Health. 2023;23(1):1006. PMID: 37254148. doi: 10.1186/s12889-023-15869-4.

4. Vujkovic B, Brkovic V, Pajičić A, Pavlovic V, Stanisavljevic D, Krajnović D, et al. Serbian version of the eHealth literacy questionnaire (eHLQ): Translation, cultural adaptation, and validation study among primary health care users. J Med Internet Res. 2024;26:e57963. PMID: 38722675. doi: 10.2196/57963.

5. Sjöström AE, Hajdarevic S, Hörnsten Å, Kristjánsdóttir Ó, Castor C, Isaksson U. The Swedish version of the eHealth literacy questionnaire: Translation, cultural adaptation, and validation study. J Med Internet Res. 2023;25:e43267. PMID: 37043268. doi: 10.2196/43267.
