# Supplementary material for: Translation and Psychometric Validation of the Amharic eHealth Literacy Questionnaire: Cross-Sectional Study
Source: J Med Internet Res. 2026 Jul 16;28:e87814. doi: 10.2196/87814 (PMC13375083; doi:10.2196/87814)
Supplement: Multimedia Appendix 2 [file jmir-v28-e87814-s002.docx]

**Multimedia Appendix**

**Table S1.** Standardized factor loadings, standard error, 95% CI, *P* value and *R*^2^ of the 7-factor model

|  |  | Standardized Factor Loading | SE | 95% CI | *P* value | *R*^2^ |
| --- | --- | --- | --- | --- | --- | --- |
| Scale 1 | eHLQ7  eHLQ11  eHLQ13  eHLQ20  eHLQ25 | 0.94  0.90  0.94  0.86  0.70 | 0.01  0.01  0.01  0.02  0.03 | 0.92 – 0.96  0.88 – 0.93  0.92 – 0.96  0.83 – 0.89  0.64 – 0.76 | <.001  <.001  <.001  <.001  <.001 | 0.88  0.81  0.88  0.74  0.49 |
| Scale 2 | eHLQ5  eHLQ12  eHLQ15  eHLQ21  eHLQ26 | 0.95  0.95  0.72  0.76  0.47 | 0.01  0.02  0.03  0.03  0.05 | 0.93 – 0.97  0.92 – 0.98  0.66 – 0.78  0.70 – 0.81  0.38 – 0.56 | <.001  <.001  <.001  <.001  <.001 | 0.90  0.91  0.52  0.57  0.22 |
| Scale 3 | eHLQ4  eHLQ6  eHLQ8  eHLQ17  eHLQ32 | 0.90  0.89  0.87  0.88  0.91 | 0.02  0.01  0.01  0.02  0.01 | 0.87 – 0.93  0.87 – 0.92  0.84 – 0.90  0.85 – 0.90  0.89 – 0.94 | <.001  <.001  <.001  <.001  <.001 | 0.81  0.80  0.76  0.77  0.83 |
| Scale 4 | eHLQ1  eHLQ10  eHLQ14  eHLQ22  eHLQ30 | 0.18  0.82  0.47  0.65  0.52 | 0.07  0.04  0.05  0.04  0.05 | 0.04 – 0.32  0.73 – 0.90  0.37 – 0.57  0.57 – 0.73  0.42 – 0.61 | 0.01  <.001  <.001  <.001  <.001 | 0.03  0.67  0.22  0.42  0.27 |
| Scale 5 | eHLQ2  eHLQ19  eHLQ24  eHLQ27  eHLQ35 | 0.88  0.92  0.85  0.86  0.86 | 0.02  0.01  0.02  0.02  0.02 | 0.85 – 0.91  0.90 – 0.94  0.82 – 0.89  0.82 – 0.89  0.82 – 0.89 | <.001  <.001  <.001  <.001  <.001 | 0.77  0.85  0.73  0.73  0.74 |
| Scale 6 | eHLQ3  eHLQ9  eHLQ16  eHLQ23  eHLQ29  eHLQ34 | 0.44  0.85  0.65  0.63  0.75  0.47 | 0.05  0.02  0.04  0.03  0.03  0.04 | 0.35 – 0.52  0.81 – 0.89  0.58 – 0.72  0.56 – 0.70  0.70 – 0.80  0.38 – 0.55 | <.001  <.001  <.001  <.001  <.001  <.001 | 0.19  0.72  0.42  0.40  0.57  0.22 |
| Scale 7 | eHLQ18  eHLQ28  eHLQ31  eHLQ33 | 0.94  0.92  0.90  0.95 | 0.01  0.01  0.01  0.01 | 0.91 – 0.96  0.89 – 0.94  0.88 – 0.93  0.93 – 0.97 | <.001  <.001  <.001  <.001 | 0.88  0.84  0.81  0.91 |
