# Supplementary material for: Translation and Psychometric Validation of the Amharic eHealth Literacy Questionnaire: Cross-Sectional Study
Source: J Med Internet Res. 2026 Jul 16;28:e87814. doi: 10.2196/87814 (PMC13375083; doi:10.2196/87814)
Supplement: Multimedia Appendix 3 [file jmir-v28-e87814-s003.docx]

**Multimedia Appendix.** Sensitivity Analysis: Confirmatory Factor Analysis Without Item 1 in Scale 4

**Table S1.** Goodness-of-fit indices of the Amharic eHLQ^a^ model without item 1

| Model | SRMR^b^ | CFI^c^ | TLI^d^ | RMSEA^e^ | Chi-square *(df)* | *P*-value |
| --- | --- | --- | --- | --- | --- | --- |
| Model without item 1 | 0.07 | 0.97 | 0.97 | 0.09 | 1698.7 (506) | <.001 |

^a^eHLQ: eHealth Literacy Questionnaire

^b^SRMR: Standardized Root Mean Residual

^c^CFI: Comparative Fit Index

^d^TLI: Tucker Lewis Index

^e^RMSEA: Root Means Square Error of Approximation

**Table S2.** Standardized factor loadings, standard error, 95% CI and *R*^2^ of the model without item 1

|  |  | Standardized Factor Loading | SE | 95% CI | *R*^2^ |
| --- | --- | --- | --- | --- | --- |
| Scale 4 | eHLQ10  eHLQ14  eHLQ22  eHLQ30 | 0.79  0.45  0.63  0.50 | 0.04  0.05  0.04  0.05 | 0.71 – 0.87  0.35 – 0.55  0.55 – 0.71  0.40 – 0.59 | 0.63  0.20  0.39  0.25 |

**Table S3.** Internal consistency (Cronbach α and McDonald ω with 95% CI) of Scale 4 of the model without item 1

|  | Cronbach α | 95% CI | McDonald ω | 95% CI |
| --- | --- | --- | --- | --- |
| Scale 4 | 0.59 | 0.47 – 0.68 | 0.70 | 0.42-0.81 |
